# Intraoperative computed tomography imaging for dose calculation in intraoperative electron radiation therapy: Initial clinical observations

**DOI:** 10.1371/journal.pone.0227155

**Published:** 2020-01-10

**Authors:** Verónica García-Vázquez, Felipe A. Calvo, María J. Ledesma-Carbayo, Claudio V. Sole, José Calvo-Haro, Manuel Desco, Javier Pascau

**Affiliations:** 1 Instituto de Investigación Sanitaria Gregorio Marañón, Madrid, Comunidad de Madrid, Spain; 2 Departamento de Oncología, Hospital General Universitario Gregorio Marañón, Madrid, Comunidad de Madrid, Spain; 3 Facultad de Medicina, Universidad Complutense de Madrid, Madrid, Comunidad de Madrid, Spain; 4 Clínica Universidad de Navarra, Madrid, Comunidad de Madrid, Spain; 5 Biomedical Image Technologies Laboratory (BIT), Escuela Técnica Superior de Ingenieros de Telecomunicación, Universidad Politécnica de Madrid, Madrid, Comunidad de Madrid, Spain; 6 CIBER-BBN, Madrid, Comunidad de Madrid, Spain; 7 Department of Radiation Oncology, Instituto de Radiomedicina, Santiago, Región Metropolitana de Santiago, Chile; 8 Servicio de Cirugía Ortopédica y Traumatología, Hospital General Universitario Gregorio Marañón, Madrid, Comunidad de Madrid, Spain; 9 Departamento de Cirugía, Facultad de Medicina, Universidad Complutense de Madrid, Madrid, Comunidad de Madrid, Spain; 10 Departamento de Bioingeniería e Ingeniería Aeroespacial, Universidad Carlos III de Madrid, Madrid, Comunidad de Madrid, Spain; 11 Centro de Investigación Biomédica en Red de Salud Mental (CIBERSAM), Madrid, Comunidad de Madrid, Spain; 12 Centro Nacional de Investigaciones Cardiovasculares Carlos III (CNIC), Madrid, Comunidad de Madrid, Spain; Dartmouth College Geisel School of Medicine, UNITED STATES

## Abstract

In intraoperative electron radiation therapy (IOERT) the energy of the electron beam is selected under the conventional assumption of water-equivalent tissues at the applicator end. However, the treatment field can deviate from the theoretic flat irradiation surface, thus altering dose profiles. This patient-based study explored the feasibility of acquiring intraoperative computed tomography (CT) studies for calculating three-dimensional dose distributions with two factors not included in the conventional assumption, namely the air gap from the applicator end to the irradiation surface and tissue heterogeneity. In addition, dose distributions under the conventional assumption and from preoperative CT studies (both also updated with intraoperative data) were calculated to explore whether there are other alternatives to intraoperative CT studies that can provide similar dose distributions. The IOERT protocol was modified to incorporate the acquisition of intraoperative CT studies before radiation delivery in six patients. Three studies were not valid to calculate dose distributions due to the presence of metal artefacts. For the remaining three cases, the average gamma pass rates between the doses calculated from intraoperative CT studies and those obtained assuming water-equivalent tissues or from preoperative CT studies were 73.4% and 74.0% respectively. The agreement increased when the air gap was included in the conventional assumption (98.1%) or in the preoperative CT images (98.4%). Therefore, this factor was the one mostly influencing the dose distributions of this study. Our experience has shown that intraoperative CT studies are not recommended when the procedure includes the use of shielding discs or surgical retractors unless metal artefacts are removed. IOERT dose distributions calculated under the conventional assumption or from preoperative CT studies may be inaccurate unless the air gap (which depends on the surface irregularities of the irradiated volume and on the applicator pose) is included in the calculations.

## Introduction

Intraoperative electron radiation therapy (IOERT) involves the delivery of a single-fraction, high-energy electron beam (4–20 MeV) to a post-resection tumour bed, which presents a high probability of harbouring residual cancer cells, or the macroscopic residue after partial resection [1]. The aim of IOERT is to promote local tumour control [2]. A specific applicator docked to a linear accelerator (LINAC) collimates the electron beam towards the treatment field. The risk of irradiating healthy tissues is reduced by displacing or protecting non-involved organs from the radiation beam [3].

In IOERT procedures, radiation oncologists choose treatment parameters according to intraoperative conditions and clinical experience. These include applicator diameter, bevel angle, applicator pose (application position and angle of beam incidence) in relation to the patient´s anatomy and prescribed dose at a specific depth. This information is transmitted to medical physicists, who select an appropriate energy of the electron beam so that a specific percentage isodose contour (commonly 90%), at which the dose is prescribed, encompasses the target volume. The beam energy is selected based on dose profiles measured in water phantoms for different energies and applicator parameters since the conventional assumption in IOERT is a flat irradiation surface with water-equivalent tissues in both stopping and scattering power at the applicator end.

A further step in IOERT dose calculation involves the use of a specific commercial treatment planning system (TPS) [4,5] or other solutions developed for this purpose [6] that take account of tissue heterogeneity by means of computed tomography (CT) studies. However, the actual treatment field can deviate from that foreseen in the TPS when using preoperative CT studies owing to variations in the patient’s position, surgical access, tumour resection and IOERT parameters. In addition, postresected surface irregularities can significantly affect the IOERT dose distribution. Costa et al [7] simulated characteristic pelvic IOERT scenarios with solid water slabs and a radiotherapy bolus, finding that a curved irradiation surface caused the two-dimensional dose distribution (measured with radiochromic films) to be curved and deeper than that with a flat irradiation surface.

Intraoperative three-dimensional (3D) images are not regularly acquired so accurate reconstruction of the irradiated volume is not available. This information is relevant to the proper assessment of clinical results [8]. Underdosage or overdosage of target volumes and organs at risk may lead to inappropriate rates of local recurrence or adverse effects. Trifiletti et al [9] pointed out several limitations of intraoperative radiation therapy in breast cancer, including the lack of intraoperative imaging to calculate customised 3D dose distributions before radiation delivery. In a preliminary experience presented at ASTRO [10], our group evaluated the difference between the 3D dose distributions when calculated from preoperative and intraoperative CT images. It was necessary to apply several preprocessing steps to preoperative images (namely virtual removal of the tumour and its surrounding tissues as performed during surgery, plus deformable registration to align preoperative and intraoperative studies) to obtain an average difference in dose of 5%. Dose distributions were calculated with Pencil Beam algorithm (method with known limitations [5]). In addition, that evaluation did not consider the distance-to-agreement concept included in the gamma index [11], which is the mainstay of comparisons between dose distributions in medical physics.

This patient-based study explored the feasibility of acquiring intraoperative CT studies for calculating IOERT 3D dose distributions, estimated with a Monte Carlo method [12], with two factors not included in the conventional assumption, namely the air gap from the applicator end to the irradiation surface (which depends on the surface irregularities of the irradiated volume and on the applicator pose) and tissue heterogeneity. This article extended the number of cases and disease sites presented in our initial report [10]. In addition, dose distributions under the conventional assumption of water-equivalent tissues at the applicator end and from preoperative CT studies (both also updated with intraoperative data) were calculated to explore whether there are other alternatives to intraoperative CT studies that can provide similar dose distributions. To our knowledge, no previous studies have pursued these objectives.

## Materials and methods

In this section, we describe the cases evaluated in this study (subsection “Cases”), the protocol followed to acquire the preoperative and intraoperative CT images (subsection “Protocol”), the processing steps applied to the images (subsection “Image processing”), the calculation of the IOERT dose distributions, and the methodology for the dose comparison (subsection “IOERT dose distributions”).

### Cases

Six patients undergoing IOERT were enrolled for this study after giving informed consent. The study was conducted in accordance with The Code of Ethics of the World Medical Association (Declaration of Helsinki) and was approved by the Ethics Committee at Hospital General Universitario Gregorio Marañón. The diagnosis of each patient and the IOERT parameters are detailed in Table 1. In both breast cancer cases, a shielding disc made of lead (diameter of 6 cm and thickness 3 mm) was used to protect intrathoracic organs during irradiation. Surgical retractors made of stainless steel were used in the retroperitoneal sarcoma case.

**Table 1:** IOERT data and CT acquisition parameters.

	IOERT DATA				CT ACQUISITION PARAMETERS		
					(In all acquisitions, voltage 120 kVp)		
	**Applicator diameter (cm)**	**Bevel angle**	**Energy (MeV)**	**90% isodose (Gy)**	**Exposure (mAs)**	**Voxel size (mm)**	**Days between CT studies**
**Patient 1**	8	15°	6	10	35 ± 7^a^^b^	1.3 x 1.3 x 5.0^b^	6
**(Ewing sarcoma)**					125^c^	1.3 x 1.3 x 2.0^c^	
**Patient 2**	12	30°	8	12.5	125	1.1 x 1.1 x 2.0^b^	1
**(Rhabdomyosarcoma)**						1.6 x 1.6 x 2.0^c^	
**Patient 3**	5	30°	6	10	100^b^	0.6 x 0.6 x 1.6^b^	17
**(Breast cancer, right)**					127 ± 66^a^^c^	1.3 x 1.3 x 3.0^c^	
**Patient 4**	5	0°	6	10	100^b^	0.7 x 0.7 x 1.6^b^	9
**(Breast cancer, left)**					114 ± 27^a^^c^	1.1 x 1.1 x 5.0^c^	
**Patient 5**	10	30°	8	12.5	196 ± 22^a^^b^	0.7 x 0.7 x 1.0^b^	1
**(Retroperitoneal sarcoma)**					217 ± 7^a^^c^	1.4 x 1.4 x 2.0^c^	
**Patient 6**	7	30°	9	12.5	132 ± 62^a^^b^	1.2 x 1.2 x 2.0^b^	0
**(Chondrosarcoma)**					143 ± 54^a^^c^	0.9 x 0.9 x 2.0^c^	

^a^Mean ± standard deviation.

^b^Preoperative CT image.

^c^Intraoperative CT image.

### Protocol

Preoperative CT images were acquired on a Toshiba Aquilion™ Large Bore CT simulator (Patients 1, 2 and 6), a Philips Mx8000 CT (Patients 3 and 4), and a Philips Brilliance-16 CT (Patient 5).

The conventional IOERT protocol included the patient transfer from the operating room (OR) to the treatment room for irradiation. This transfer was necessary, since a dedicated mobile LINAC was not available inside the OR. The IOERT protocol was modified to incorporate the acquisition of the intraoperative CT study of the actual scenario as follows:

1. The patient lay on a rigid radiotransparent subtable that was placed on the operating table during surgery.
2. After tumour resection, the IOERT applicator was placed over the tumour bed and firmly attached to the radiotransparent subtable with an articulated arm.
3. The patient was covered to maintain asepsis of the surgical field during transfer to the CT simulator room and treatment room. A subtable stretcher, similar to that presented in [13], made it possible to carry the subtable from the operating table to the CT/LINAC table. The radiotransparent subtable and the subtable stretcher were custom-made for this study.
4. An intraoperative CT image of the whole setting (applicator placed on the treatment field) was acquired on a Toshiba Aquilion™ Large Bore CT simulator.

After these steps, the conventional protocol was followed: the patient was transferred to the treatment room for irradiation with a fixed LINAC (Elekta Precise Treatment System™) and then back to the OR to complete the surgical procedure. This protocol did not include in vivo dosimetry. Patients were monitored under general anaesthesia throughout the IOERT procedure. No IOERT decisions were taken based on the intraoperative images. Table 1 shows CT acquisition parameters of preoperative and intraoperative images, and the time interval between both studies.

### Image processing

#### Image registration

Preoperative CT images were rigidly registered to their corresponding intraoperative CT studies to calculate dose distributions in the same coordinate space and with the same applicator pose in relation to the patient’s anatomy. This image processing was done by carrying out the following steps with MMWKS software [14]. First, CT images were resampled to 1.5-mm isotropic voxel size, and bone structures close to the treatment volume were segmented using a region-growing method [15] plus manually delineated boundaries. After this, preoperative images were aligned with their corresponding intraoperative images by using an automatic rigid registration algorithm based on normalised mutual information as a cost function [16], calculated only in the segmented bones.

#### Air gap segmentation

With respect to dose calculation, an important difference between preoperative and intraoperative CT images is related to the potential air gap from the applicator end to the irradiation surface (which depends on the surface irregularities of the tumour bed and on the applicator pose). This feature was obtained from the intraoperative CT images by segmenting the air gap along the longitudinal extension of the applicator using a region-growing method (maximum limit –500 Hounsfield units, HU) plus manually delineated boundaries. The air gap was included in the registered preoperative CT images by setting the corresponding segmented voxels to air value (–1000 HU). For each IOERT case, another image was created to take account of the air gap but not the tissue heterogeneities in dose comparisons by also setting the remaining voxels of each modified registered preoperative image to the water value (0 HU).

#### Identification of the applicator pose

The following semiautomatic method was used to obtain the applicator pose (position of the bevel centre and rotation of the applicator) in each intraoperative image since the CT values of the applicator, which was made of polymethyl methacrylate, are similar to those of soft tissues. First, a segmentation of the applicator wall with a region-growing method plus manually delineated boundaries was used to calculate the X and Y coordinates of the bevel centre and the geometric axis of the applicator. After this, the end of the shorter edge of the applicator was manually located at an axial slice, enabling us to find the remaining parameters of the applicator pose.

### IOERT dose distributions

IOERT cases were simulated using *radiance* TPS (GMV, Spain) [4,5], the registered preoperative and intraoperative images of each patient, and the IOERT parameters shown in Table 1. The parameters obtained in subsection “Identification of the application pose” were used to match the pose of the virtual applicator in the TPS to that depicted in each intraoperative image. The TPS automatically modified the CT value of the voxels inside the applicator, setting them to the air value.

Dose distributions were calculated based on a Monte Carlo algorithm specifically adapted for IOERT [12] (uncertainty 1%, resolution 1.5 mm), the phase space of the LINAC [17], and the registered preoperative and intraoperative images of each patient after converting HU values to physical density [12,18] as follows:

- D_intraCT, considered the gold standard as this dose distribution was calculated from each intraoperative study, which showed the actual IOERT scenario before irradiation (namely, after tumour removal and with the applicator in place).
- D_preCT_water_, dose distribution calculated from each registered preoperative image by selecting the *Water* option in the TPS to assume water-equivalent tissues at the applicator end (conventional assumption).
- D_preCT, dose distribution calculated from each registered preoperative image, thus including tissue heterogeneities but not the air gap at the applicator end.
- D_preCT_water&air_, dose distribution calculated from each registered preoperative image assuming water-equivalent tissues and including the air gap at the applicator end (as detailed in subsection “Air gap segmentation”).
- D_preCT_air_, dose distribution calculated from each registered preoperative image (thus taking into account tissue heterogeneities) and including the air gap at the applicator end (as detailed in subsection “Air gap segmentation”).

D_PreCT_water_, D_preCT, D_preCT_water&air_ and D_preCT_air_ were compared with the corresponding D_intraCT using a 3D gamma criteria of 3%/3 mm for dose values greater than 10% or 70% (to focus on high-dose regions) [18], and global normalisation [19]. These comparisons were intended to identify the relative contribution in dose calculation of tissue heterogeneity and air gap (which depended on the surface irregularities of the tumour bed and on the applicator pose) in order to explore whether there are other alternatives to intraoperative CT studies. Voxels not belonging to patient tissue in each D_intraCT were not taken into account in the 3D gamma analyses.

## Results

The six patients in this study underwent IOERT following the protocol described. There were no complications for the patients during transfer, which was performed using the subtable and its stretcher, or during the acquisition of intraoperative CT images (anaesthetic instability or any other relevant clinical observations). This protocol was applied by a coordinated multidisciplinary team that was accustomed to performing the conventional protocol, which involved transferring the anaesthetised patient from the OR to the treatment room for irradiation.

**Fig 1:**
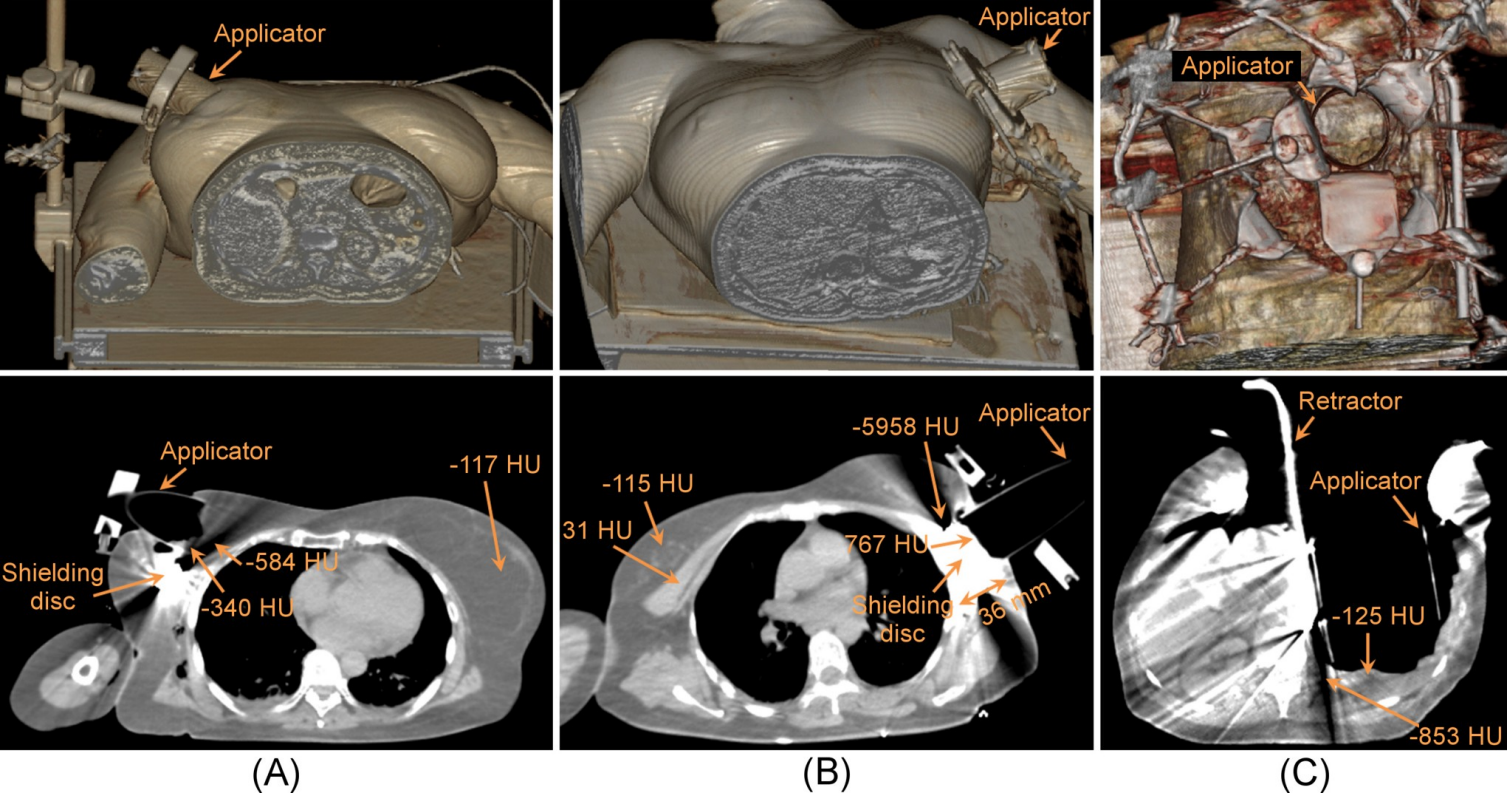
Intraoperative images with metal artefacts. Volume rendering view (top) and axial view (bottom). (A) Patient 3 (breast cancer, right). (B) Patient 4 (breast cancer, left). (C) Patient 5 (retroperitoneal sarcoma).

Dose distributions were not calculated in three cases (Patients 3, 4, and 5) owing to artefacts (severe streaking) in the intraoperative images caused by metallic objects (shielding discs in the breast cancer cases and surgical retractors in the retroperitoneal sarcoma case), which substantially modified the CT values (Fig 1). These intraoperative images were not appropriate to calculate gold standards for dose comparisons. In both breast cancer cases (Fig 1A and 1B), there were dark streaks along the radial axis of the shielding discs and bright streaks along its perpendicular axis. These artefacts were larger than the dimensions of the shielding disc. In the case of Patient 3, the applicator was not sufficiently firmly attached and moved during the transfer to the CT room. This attachment problem was detected at the treatment room where the applicator pose was corrected before irradiation. The intraoperative image showed that the shielding disc was not aligned with the applicator (Fig 1A).

**Fig 2:**
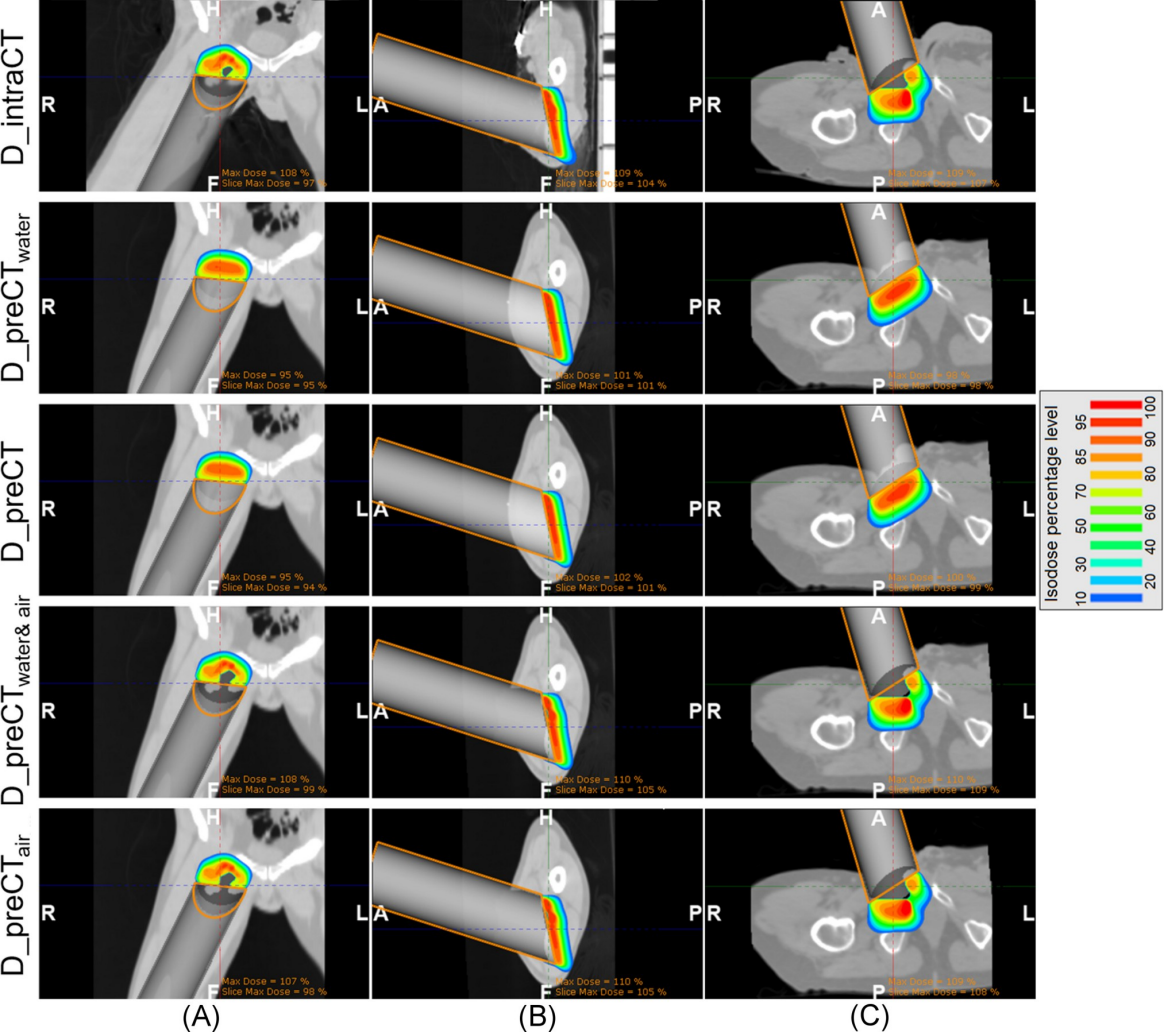
Dose distributions calculated using the Monte Carlo algorithm. (A) Patient 1 (Ewing sarcoma, coronal view). (B) Patient 2 (rhabdomyosarcoma, sagittal view). (C) Patient 6 (chondrosarcoma, axial view). H (head), F (feet), A (anterior), P (posterior), R (right) and L (left).

Registration between the preoperative and intraoperative CT studies (specifically the bone structures close to the treatment volume) was checked by visual inspection. As expected, the alignment of structures far from the treatment volume was not perfect owing to the rigid transformation used for registration but inside the treatment volume it was correct. The root-mean-square difference between both images and considering just voxels belonging to patient tissue was 88 HU (Patient 1), 95 HU (Patient 2) and 113 HU (Patient 6). The volumes of interest of these measurements were limited to those of the 3D dose distributions (Table 2). Voxels not belonging to patient tissue in each D_intraCT were not taken into account as set in the 3D gamma analyses. The maximum distances from the applicator end to the surface of the tumour bed were 14.5 mm (Patient 1), 8.5 mm (Patient 2) and 30.5 mm (Patient 6). Dose distributions are shown in Fig 2 (Patients 1, 2 and 6). The average gamma pass rates were 73.4% and 74.0% for D_preCT_water_ and D_preCT respectively (Table 2). Better results were found when the air gap was included in the IOERT dose calculation (98.1% and 98.4% for D_preCT_water&air_ and D_preCT_air_ respectively).

**Table 2:** Percentage of voxels fulfilling 3D gamma criteria of 3%/3 mm. Gamma pass rates ≥ 95% highlighted in bold.

		Dose > 10%	Dose > 70%
**Patient 1**	D_intraCT v D_preCT_water_	81.9	81.5
	D_intraCT v D_preCT	81.8	81.6
**(Ewing sarcoma)**	D_intraCT v D_preCT_water&air_	**99.8**	**99.9**
	D_intraCT v D_preCT_air_	**99.5**	**99.8**
**Patient 2**	D_intraCT v D_preCT_water_^a^	90.0	92.1
	D_intraCT v D_preCT	92.3	92.2
**(Rhabdomyosarcoma)**	D_intraCT v D_preCT_water&air_	**97.3**	**99.3**
	D_intraCT v D_preCT_air_	**99.6**	**99.4**
**Patient 6**	D_intraCT v D_preCT_water_^b^	52.6	42.0
	D_intraCT v D_preCT	53.6	42.5
**(Chondrosarcoma)**	D_intraCT v D_preCT_water&air_	**95.0**	**97.5**
	D_intraCT v D_preCT_air_	94.5	**97.8**

Dose matrices: 183 x 235 x 175, 180 x 156 x 256 and 200 x 179 x 250 for Patient 1, Patient 2 and Patient 6 respectively. Voxel size 1.5 x 1.5 x 1.5 mm.

Average gamma pass rates: 73.4%, 74.0%, **98.1**% and **98.4**% for D_preCT_water_, D_preCT, D_preCT_water&air_ and D_preCT_air_ respectively.

^a^Average gamma pass rate: 91.1%.

^b^Average gamma pass rate: 47.3%.

## Discussion

This is the first patient-based study that explores the feasibility of acquiring intraoperative CT studies for calculating IOERT 3D dose distributions, apart from our initial report presented at ASTRO [10]. There were no complications for the patients during the modified IOERT protocol that incorporated the acquisition of the actual scenario before irradiation. These images also allow inspection of the protection assembly (Fig 1A and 1B), which is the major source of problems in IOERT [20] because of the lack of direct visual inspection of the shielding disc. However, standard clinical practice cannot include the use of a CT simulator for intraoperative imaging in IOERT. Transferring the patient to the CT simulator room involves additional risks for the patient and may not be indicated when a dedicated mobile LINAC is available inside the OR. Other possible ways to acquire these intraoperative images for calculating IOERT 3D dose distributions are a portable CT inside the OR, or even a LINAC that includes on-board kV cone beam CT [18]. However, a low number of treatments per week may not justify the installation costs of in-room imaging [21].

Intraoperative CT imaging in IOERT procedures allows the calculation of 3D dose distributions with two factors not included in the conventional assumption, namely the air gap from the applicator end to the irradiation surface (which depends on the surface irregularities of the irradiated volume and on the applicator pose) and tissue heterogeneity. The main limitation of these intraoperative images for dose calculation is the presence of metal artefacts in some IOERT scenarios, owing to shielding discs and surgical retractors. A two-layered disc such as the one described in [22] would decrease, but not remove, the metal artefacts, since copper has a lower attenuation coefficient than lead. In the case of the retractors, a possible approach would be to use a nonmetallic version, although this is not common in clinical practice. The solution presented in [23] was a 3D-printed thermoplastic Army/Navy retractor. An alternative would be to replace incorrect CT values with the corresponding ones from the registered preoperative image or to include a metal artefact reduction algorithm in CT reconstruction [24], although this would require further research on assessing the accuracy of the IOERT dose distributions after applying those corrections.

Surface irregularities influenced doses, as displayed in the 3D dose distributions obtained from the intraoperative images (Fig 2A and 2C). These results were expected given the findings from previous studies [7,25] (phantom study and in vivo study, respectively), where two-dimensional dose distributions were obtained from radiochromic films placed on the irradiation surface. In [25], the authors showed that measured doses frequently differ from the expected ones in IOERT of rectal cancer (scenario that often presents surface irregularities). The approach used in our study was to calculate 3D dose distributions of the actual IOERT scenario before irradiation, instead of measuring a partial view of the dose distribution with radiochromic films, showing interesting spatial details about the dose distribution. For instance, D_intraCT corresponding to Patient 6 (Fig 2C) presented a hot spot probably due to the scatter produced by the sharp irregularities on the irradiated surface [26], and a flat irradiation surface probably related to the accumulation of biological fluid in that region, which affects the dose distribution (build-up effect) [25,27]. On the other hand, the implemented Monte Carlo algorithm has some limitations since it does not take account of tissue bulging into the applicator opening, as shown in Fig 2.

Despite the reduced number of patients in our study, three different situations in terms of dose agreement were found, ranging from one where assuming water-equivalent tissues was almost valid (Patient 2) to other where the conventional assumption provided inaccurate dose distributions (Patient 6). In the evaluated IOERT scenarios, the greater the maximum distance from the applicator end to the surface of the tumour bed (air gap), the larger the difference in gamma criteria between D_preCT_water_ and D_intraCT. The best case corresponded to Patient 2 (maximum distance of 8.5 mm and an average gamma pass rate of 91.1%) while the worst case corresponded to Patient 6 (maximum distance of 30.5 mm and an average gamma pass rate of 47.3%).

Registered preoperative images did not improve the calculation of dose distribution (D_preCT, average gamma pass rate of 74.0%) compared with the usual IOERT simplification (D_preCT_water_, average of 73.4%), even though those images included tissue heterogeneities and the information regarding the applicator pose in relation to the patient’s anatomy. A better dose agreement was found when the air gap was included in the conventional assumption (D_preCT_water&air_, average of 98.1%) or in the preoperative images (D_preCT_air_, average of 98.4%, approach that takes into account of tissue heterogeneity). Therefore, the air gap was the factor mostly influencing the dose distributions of this study with a different impact depending on the IOERT case (namely, less impact in Patient 2 than in Patient 6). Previous studies have reported different scenarios regarding the air gap: lengths up to 5 cm in soft-tissue sarcomas of distal limbs [28], irregular and/or concave surfaces in rectal cancer [25], and soft breast tissue adapted to the flat end of a non-bevelled applicator [7]. Therefore, air gap should be included in IOERT records to assess whether the conventional assumption is valid in each treatment. Finally, tissue heterogeneity was not a key factor in the cases evaluated since bones were at a certain distance from the tumour bed, but it would be the case in rectal cancer since the tumour bed or high-risk area is very close to the sacrum.

The acquisition of the actual IOERT scenario before irradiation, specifically the surface irregularities of the tumour bed and the applicator pose, is relevant to record the treatment administered to the patient. Surface scanning of the irradiated volume combined with the applicator pose and assuming water-equivalent tissues from the irradiation surface, as proposed in [29], might be an alternative to explore in the future. Nevertheless, including this approach in the IOERT workflow entails first addressing some practical problems. Further research on improving the accuracy of the IOERT dose calculation is commended since these dose distributions would allow assessment of the treatment outcome and calculation of dose accumulation when external beam radiation therapy is applied with an IOERT boost component.

## Conclusions

Intraoperative CT studies for calculating 3D dose distributions were acquired in several IOERT scenarios with no complications for the patients. Our experience has shown that intraoperative CT studies are not recommended when the procedure includes the use of shielding discs or surgical retractors unless metal artefacts are removed. Air gap and not tissue heterogeneity was the factor mostly influencing the evaluated dose distributions. The conventional assumption of water-equivalent tissues at the applicator end or the use of preoperative CT studies may lead to inaccurate IOERT dose distributions unless the air gap from the applicator end to the irradiation surface (which depends on the surface irregularities of the irradiated volume and on the applicator pose) is included in the calculations.
